# Supplementary material for: Content-rich biological network constructed by mining PubMed abstracts
Source: BMC Bioinformatics. 2004 Oct 8;5:147. doi: 10.1186/1471-2105-5-147 (PMC528731; doi:10.1186/1471-2105-5-147)
Supplement: Additional File 2 — The original results of the above study (non-essential files are deleted to keep the file size under the limit set by BMC bioinformatics). [file 1471-2105-5-147-S2.bz2 › chilibotAdditionalFile2/dip30/351ID8663118E4684/html/IL13_IL13RA2.html]

 


 **IL13** and **IL13RA2** 
  
Found 82 abstracts in PubMed, retrieved 30.  
 

 What does Google say? 
 PDF only 
| .edu only 

---

**Interactive relationship** (e.g. stimulation, inhibition, etc)

**Neutral relationship**- IL 13  [ **IL13** ]  showed a modest antitumor activity to IL 13R  [ **IL13RA2** ]  alpha 2 chain overexpressing tumors in vitro and in vivo.  Ref: 11748276 J Exp Med, 2001
- Interleukin 13  [ **IL13** ]  receptor IL 13R  [ **IL13RA2** ]  targeted cytotoxin,  **IL13**  PE38QQR, composed of IL 13  [ **IL13** ]  and a mutated form of Pseudomonas exotoxin PE, is found to be highly and specifically cytotoxic to human solid cancer cell lines.  Ref: 11862421 Cancer Immunol Immunother, 2002
- Our third generation experiments suggest that this approach is feasible for clinical situations as intratumor administration of plasmid carrying the IL 13R  [ **IL13RA2** ]  alpha2 chain gene sensitized these tumors to systemic or regional  **IL13**  PE therapy.  Ref: 12187325 Anticancer Drugs, 2002
- RT PCR showed that each culture expressed IL 4R alpha, common gamma chain, and IL 13R  [ **IL13RA2** ]  alpha 1, as well as IL 13R  [ **IL13RA2** ]  alpha 2, which negatively regulates IL 13  [ **IL13** ]  signaling.  Ref: 12077271 J Immunol, 2002
- Thus, IL 13  [ **IL13** ]  cytotoxin may be a useful agent for high IL 13R  [ **IL13RA2** ]  expressing SCCHN.  Ref: 12060640 Clin Cancer Res, 2002
- IL 13  [ **IL13** ]  exerts its actions by binding to the IL 13  [ **IL13** ]  receptor IL 13R  [ **IL13RA2** ]  on target cells, which is composed of IL 13Ralpha1 and the IL 4 receptor alpha chain IL 4Ralpha.  Ref: 11145846 Cytokine, 2001
- Acquisition of sensitivity to  **IL13**  PE was observed both in vitro and in vivo when IL 13R  [ **IL13RA2** ]  alpha2 transfected human tumor cells were implanted in immunodeficient animals followed by systemic or regional  **IL13**  PE therapy.  Ref: 12187325 Anticancer Drugs, 2002
- IL 13  [ **IL13** ]  receptor alpha chain 1 IL 13R  [ **IL13RA2** ]  alpha 1 is necessary for binding to IL 13  [ **IL13** ] , and the heterodimer composed of IL 13R  [ **IL13RA2** ]  alpha 1 and IL 4R alpha chain transduces IL 13  [ **IL13** ]  and IL 4 signals.  Ref: 11058569 Int Immunol, 2000
- IL 13  [ **IL13** ]  cytotoxin, composed of IL 13  [ **IL13** ]  and a truncated form of Pseudomonas exotoxin, targets IL 13R  [ **IL13RA2** ]  overexpressing tumor cell lines in vitro and in vivo.  Ref: 12455052 Int J Cancer, 2003
- BACKGROUND IL 13  [ **IL13** ]  receptor IL 13R  [ **IL13RA2** ]  targeted cytotoxin,  **IL13**  PE38QQR, has been shown to have very potent anti tumor activity to IL 13R  [ **IL13RA2** ]  expressing head and neck tumor cells in vitro and in vivo.  Ref: 12435859 Mol Med, 2002
- In contrast, normal human astrocytes that did not express the IL 13R  [ **IL13RA2** ]  alpha 2 gene efficiently induced Stat6 activation in response to both IL 4 and IL 13  [ **IL13** ] .  Ref: 11861389 Cancer Res, 2002
- This was in part because of the expression of a high affinity IL 13  [ **IL13** ]  binding transmembrane protein IL 13R  [ **IL13RA2** ]  alpha 2 that inhibited IL 13  [ **IL13** ]  mediated Stat6 activation by acting as a decoy receptor.  Ref: 11861389 Cancer Res, 2002
- Interleukin IL 13  [ **IL13** ]  and IL 4 inhibit proliferation and stimulate IL 6 formation in human osteoblasts evidence for involvement of receptor subunits IL 13R  [ **IL13RA2** ] , IL 13Ralpha, and IL 4Ralpha.  Ref: 11248656 Bone, 2001

**Non-interactive relationship** (e.g. studied together, co-existance, homology, etc.)

- The interleukin 13  [ **IL13** ]  receptor IL 13R  [ **IL13RA2** ]  complex is composed of 2 different chains, IL 13Ralpha1 also known as IL 13Ralpha and IL 13Ralpha2 also known as IL 13Ralpha.  Ref: 11313257 Blood, 2001
- IL 13R  [ **IL13RA2** ]  alpha 2, a decoy receptor for IL 13  [ **IL13** ]  acts as an inhibitor of IL 4 dependent signal transduction in glioblastoma cells.  Ref: 11861389 Cancer Res, 2002
- H RS cells of MC and NS types frequently expressed both IL 13  [ **IL13** ]  and IL 13R  [ **IL13RA2** ] .  Ref: 11318902 Histopathology, 2001
- it also acts as the signaling chain for IL 13  [ **IL13** ] , but in this case the IL 13R  [ **IL13RA2** ]  alpha 1 chain conveys the bulk of the cytokine specificity.  Ref: 12165974 Roum Arch Microbiol Immunol< MedlineTA>Roum Arch Microbiol Immunol, 2001
- Although some prostate cancer cell lines express functional IL 13R  [ **IL13RA2** ] , they are not highly sensitive to IL 13  [ **IL13** ]  cytotoxin.  Ref: 11773976 Cancer Gene Ther, 2001
- To study the function of IL 13R  [ **IL13RA2** ]  on brain tumor cells, we investigated the regulation of adhesion molecules by IL 13  [ **IL13** ]  as assessed by flow cytometric analysis.  Ref: 11939409 Oncol Res, 2000
- These receptors are composed of IL 13  [ **IL13** ]  receptor IL 13R  [ **IL13RA2** ]  alpha1, IL 13Ralpha2, and IL 4Ralpha chains.  Ref: 11939409 Oncol Res, 2000
- Expression of IL 13R  [ **IL13RA2** ]  alpha 1 evoked STAT3 activation by IL 4 and IL 13  [ **IL13** ] , and in stimulated human B cells, on which IL 13R  [ **IL13RA2** ]  alpha 1 was highly expressed, IL 4 and IL 13  [ **IL13** ]  induced STAT3 activation.  Ref: 11058569 Int Immunol, 2000
- The inability of BL30 B95 8 cells to respond to IL 13  [ **IL13** ]  was attributed to the loss of expression of IL 13R  [ **IL13RA2** ]  subunits alpha1 and alpha2, a finding confirmed for a number of other BL cell lines examined.  Ref: 11591117 Cell Immunol, 2001
- Therefore, additional studies should be performed to determine the expression of IL 13  [ **IL13** ]  and IL 13R  [ **IL13RA2** ]  in primary clinical samples of Hodgkin s lymphoma and both agents should be further tested in vitro and in vivo as possible therapeutic agents for HL.  Ref: 11585386 Cell Immunol, 2001
- METHODS AND RESULTS The expression of IL 13  [ **IL13** ]  and IL 13  [ **IL13** ]  receptor IL 13R  [ **IL13RA2** ]  was determined by immunostaining and reverse transcriptase polymerase chain reaction in 39 cases of HD, including 17 cases with nodular sclerosis NS type, 19 cases with mixed cellularity MC, and three cases with lymphocyte predominance LP type.  Ref: 11318902 Histopathology, 2001
- Transient expression of the IL 13R  [ **IL13RA2** ]  alpha 2 transgene in nonexpressing heterologous cells inhibited not only IL 13  [ **IL13** ]  but also IL 4 mediated signal transduction and Stat6 responsive gene expression.  Ref: 11861389 Cancer Res, 2002
- To elucidate the mechanism, we utilized four head and neck cancer cell lines SCC 25, HN12, KCCT873, and YCUM911, which express high levels of IL 13R  [ **IL13RA2** ] , and IL 13  [ **IL13** ]  toxin is highly cytotoxic to these cells.  Ref: 11862421 Cancer Immunol Immunother, 2002
- We have previously reported that interleukin 13  [ **IL13** ]  receptors IL 13R  [ **IL13RA2** ]  are constitutively overexpressed on a majority of human malignant glioma cell lines and primary cell cultures.  Ref: 12481422 Mol Cancer Ther, 2002
- In this study, we constructed wild and mutated types of human IL 13R  [ **IL13RA2** ]  alpha 1, and analyzed IL 4 and IL 13  [ **IL13** ]  signals using an IL 13R  [ **IL13RA2** ]  alpha 1 transfected human B cell line.  Ref: 11058569 Int Immunol, 2000
- Interleukin 13  [ **IL13** ]  receptor alpha2 IL 13R  [ **IL13RA2** ]  alpha 2 chain is highly expressed on some tumor cell lines and primary cell cultures.  Ref: 11748276 J Exp Med, 2001
- Inflammatory cells including neutrophils and macrophages were identified in IL 13R  [ **IL13RA2** ]  alpha 2 overexpressing regressing tumors and neutrophils were found to produce IL 13  [ **IL13** ] .  Ref: 11748276 J Exp Med, 2001
- However, the number of IL 13  [ **IL13** ]  positive H RS cells was statistically higher in NS type than in MC type, but the number of IL 13R  [ **IL13RA2** ]  was similar.  Ref: 11318902 Histopathology, 2001
- Although interleukin 13  [ **IL13** ]  receptors IL 13R  [ **IL13RA2** ]  are overexpressed on several head and neck cancer cell lines, a majority of cell lines express only low levels of IL 13R  [ **IL13RA2** ] .  Ref: 11507072 Cancer Res, 2001
- Several HL derived cell lines expressed IL 13  [ **IL13** ]  message and protein and message for various chains of IL 13R  [ **IL13RA2** ] .  Ref: 11585386 Cell Immunol, 2001
- We have demonstrated that when interleukin IL 13  [ **IL13** ]  receptor IL 13R  [ **IL13RA2** ]  alpha2 chain, one of the two known IL 13  [ **IL13** ]  binding proteins, is introduced into cancer cells that do not express this chain the cells acquire extreme sensitivity to a chimeric fusion cytotoxin composed of IL 13  [ **IL13** ]  and a mutated form of Pseudomonas exotoxin  **IL13**  PE.  Ref: 12187325 Anticancer Drugs, 2002
- IL 13  [ **IL13** ]  and IL 13R  [ **IL13RA2** ]  alpha 1, the IL 13  [ **IL13** ]  specific receptor chain, are frequently expressed by HL derived cell lines and by RS cells from biopsy material of tissues involved by HL.  Ref: 11815733 Int Arch Allergy Immunol, 2001
- Here, we report that Jak2 is associated with IL 4R alpha, and Tyk2 is associated with the IL 13R  [ **IL13RA2** ]  alpha 1 component of the IL 13  [ **IL13** ]  receptor complex.  Ref: 12223527 J Leukoc Biol, 2002
- This was followed by successfully treating xenograft tumor bearing nude mice with IL 13R  [ **IL13RA2** ]  directed cytotoxin  **IL13**  PE38QQR.  Ref: 12471149 J Immunol, 2002
- Interleukin 13  [ **IL13** ]  receptor IL 13R  [ **IL13RA2** ]  alpha2 chain plays a key role in ligand binding and internalization.  Ref: 12808442 Gene Ther, 2003
- Coexpression of IL 13  [ **IL13** ] , IL 13R  [ **IL13RA2** ]  alpha 1 and P STAT6 is uncommon in non Hodgkin lymphomas.  Ref: 11815733 Int Arch Allergy Immunol, 2001
- IL 13  [ **IL13** ]  and the IL 13  [ **IL13** ]  specific receptor chain IL 13R  [ **IL13RA2** ]  alpha1 are frequently expressed in cHL derived cell lines and in RS cells from biopsies of cHL tissues.  Ref: 12152987 Leuk Lymphoma, 2002
